# Augusta Academy of Medicine

**Published:** 1894-12

**Authors:** 


### *SOCIETY REPORTS.*

### AUGUSTA ACADEMY OF MEDICINE.

Augusta, Ga., October 3, 1894.

*The President, Dr. Jos. Eve Allen, in the Chair.*

Discussion on the subject of the

### LICENSING OF MIDWIVES.

The committee appointed at the last meeting to consider and report upon the matter of the licensing of midwives reported progress, through its chairman, Dr. J. E. Allen, and requested that the subject be further discussed by the Academy. He stated that the committee had in preparation a bill to be presented to the legislature, after its indorsement by the Academy, which provided that midwives practicing in Richmond county should be required to take out a license with the ordinary; the ordinary to grant the license upon the presentation of a certificate signed by three reputable physicians, stating that the midwife is, in their opinion, from examination and observation, capable of managing an ordinary normal case of labor. She must also present a certificate from some clergyman, setting forth that she is of good moral character.

The chairman said that this was a very important matter, and demanded the attention of the Academy. He was not in favor of doing away with midwives; this could never be done. What was necessary was to protect the public by restricting the practice to those who were competent to render proper service. The licensing of midwives was now under discussion in the medical societies of New York and Philadelphia; and the British Medical Association was also considering the matter.

Dr. Eugene Foster said that the midwife was a necessary evil, and relieved the physician of much work that was entirely gratuitous. He was not in favor of making them conform to requirements that at present they could not meet. In this city there were no facilities for instructing midwives unless the Augusta Training School for Nurses proposed to make this branch a part of its curriculum.

DR. JAMES B. MORGAN said that he believed that the duty of a physician did not stop at the mere curing of diseases, but that he was also the conservator of public health and morals. The present practice of midwifery by midwives was full of danger to the community, and great and unnecessary suffering and death resulted from it. The only cases of puerperal fever that had ever come under his observation occurred in the practice of midwives. As coroner's physician he was often called upon to examine still-born infants coming from the same source. He was satisfied that this was almost always due to the lamentable want of knowledge and skill of the midwives of this city. He stated that three States of the Union had laws regulating the practice of midwives. He thought it just as necessary to throw legal safeguards around midwifery practice as it was to require doctors, druggists, and dentists to be licensed. In Germany and France, where the midwives were educated, the percentage of still births was very small, amounting to only three or four per cent. In New York, where there is no educational requirement, but only a license is necessary to practice, the per cent. is eight or nine. He was satisfied that the per cent. here was much greater, but could not obtain reliable statistics, owing to the fact that the Augusta Board of Health did not demand the record of still births.

DR. H. H. MALONE stated that he thought that the licensing of midwives, without requiring of them sufficient training, would have the bad effect of giving to certain ones undue prominence, which they did not deserve, and which would enable them to impose upon the public. He was opposed to giving midwives any legal recognition until they could be properly educated. He asked if the law as proposed by the committee passed, if a woman who came in in an emergency in the absence of a physician, or licensed midwife, and assisted a woman in labor, would not be violating the law and be liable to punishment.

DR. J. E. ALLEN said that a woman who, in an emergency, assisted a woman in labor, when a doctor or midwife could not be had, would not be violating the law any more than a man would be who, seeing a person bleeding to death in the street from a wound of the femoral artery, ties a band around the limb and stops the hemorrhage.

DR. WM. B. CRAWFORD then presented a paper upon "The Aseptic and Antiseptic Treatment of Wounds." (See page 603.)

THE PRESIDENT announced the discussion open, and said that he had nothing to say as to the surgical aspect of asepsis or antisepsis, but he wished to say, in justice to the branch of practice in which he was directly interested, that in obstetrics the importance of these measures were recognized and advocated twenty years before Lister first called the attention of surgeons to their value. In 1847 Semmelweiss made the great discovery that puerperal fever was really nothing more nor less than septicemia, the result of the infection of the genital tract either by the dirty hands or instruments of the accoucheur or nurse, or by the retention of decomposing material, such as blood-clots, shreds of membrane, pieces of placenta, etc. Semmelweiss insisted upon the observance of thorough asepsis in the management of labor cases, and his teachings have resulted in stamping out puerperal fever almost altogether. Now large maternities offer the safest conditions for delivery, septicemia being met with usually in private practice, where strict asepsis cannot be enforced.

The obstetrician to-day prepares his patient for labor with the same careful attention to absolute cleanliness in every particular that the surgeon observes in getting his patient ready to undergo laparotomy or any other capital operation.

DR. W. H. DOUGHTY, Jr., stated that he had listened to the paper read by the essayist with a great deal of pleasure, and that he heartily indorsed most of the statements presented therein. In regard to the sterilizing of instruments, he did not consider it necessary to employ both dry heat and boiling in solution of carbonate of soda to effect asepsis. Either of these methods alone would be sufficient. He generally preferred boiling in soda solution. The most difficult part of the aseptic technique was to get the skin surgically clean. This was done by the use of certain gerimcidal agents, such as carbolic acid, bichloride of mercury, etc. Some surgeons preferred one agent, some another. In his opinion it did not matter what antiseptic was used, so the cleansing process was thoroughly performed. The point to be gained was the exclusion of the pathogenic germs, and it was of no consequence whether we kept them out by antiseptics or built a wall around the patient and in that way kept them out. As an antiseptic, he most frequently used carbolic acid, because it was more managable and did not affect the plating of instruments like the bichloride solutions. He also objected to the bichloride because it makes the hands rough. For use within the abdominal cavity simple boiled water was preferred, and was fully sufficient for ordinary asepsis. In the treatment of lacerated wounds he had found that healing was often interfered with by the dressings adhering to the wound; to prevent this he always, after thoroughly cleansing, first applied a piece of gutta-percha tissue, and over this put the gauzes and other dressings. To clean contused and lacerated wounds he employed a strong solution of crystal carbolic acid in glycerine; this killed the germs and rendered the parts anesthetic. To close granulating wounds he passed sutures not through the sensitive external layers of the skin, but below, and thus was often able to painlessly bring together large wounds without the use of an anesthetic.

DR. EUGENE FOSTER said that soap and water was the antiseptic agent advocated by Semmelweiss in obstetric practice, and with it as good results can be obtained in surgery as by the employment of cumbrous and complicated methods. He thought that Lister had received much more credit than he deserved, for the methods that he first practiced, if followed to-day, would subject a surgeon to prosecution in the courts for malpractice. Dr. Foster did not regard Lister as the father of modern surgery. Asepsis is in no sense Listerism as originally taught by him. Listerism is disinfectant surgery, and not the aseptic surgery of to-day. The farther surgeons have departed from Listerism, the better have been their results. Aseptic surgery was as fully and as intelligently practiced and taught twenty-five years ago by Professor L. A. Dugas, of the University of Georgia, as is the aseptic surgery of to-day.

DR. THEODORE LAMB said, probing properly done did no harm, and gave definite information instead of guesswork. Whenever a bullet was lodged in firm tissues, as in the limbs, the bullet track should first be thoroughly irrigated with an antiseptic solution. A long, small caliber injector should be inserted to bottom of track and a brisk regurgitant current used. This mechanically removes gross particles, and also renders track aseptic. Probe cautiously and systematically, and *be done with it at the first sitting;* follow with strict antiseptic dressings. Aseptic bullets often produce secondary mischief—viz., prolonged suffering, traumatic aneurism, and waste of valuable time. Septic particles may easily be carried to same depth as bullet; can never be sure they are not present; if present, leaving the bullet multiplies the risks tenfold. Therefore probe and remove bullet wherever feasible, *but do both properly.* A perfectly clean probe in an infected bullet track is sure to do mischief.

As to vaginal irrigations in normal parturition, he did not believe them necessary; but whenever suppurative inflammation exists in the genital tract, it demands antiseptic treatment. As for instance, gonorrheal vaginitis, etc.

DR. C. J. MONTGOMERY said: Granting that, from a clinical point of view, pyogenic microbes were the etiological factor in the production of suppuration, the question as to its direct cause should be considered; whether it could be produced without those microbes, and under what circumstances those microbes failed to produce it. He then referred to the views of Cheyne, that the real cause was the ferment produced by the action of those micro-organisms, or at least some chemical substance was necessary, which prevented the coagulation of the exuded fluid. He referred to suppuration having been produced by the sterilized cultures of micro-organisms of suppuration; and also to the experiments of Grawitz, Councilman, and others, who had produced abscesses by the injection of sterilized turpentine and croton oil, the pus from the resulting abscesses being subsequently shown to be free from micro-organisms. He also called attention to Passet's experiment of inserting a needle into a culture of pus microbes, and then inserting this needle into joints failing to produce suppuration, but if the pus microbes were first mixed with water, and this injected into a joint, suppuration readily took place. He referred also to the experiments of Orth, Grawitz, and others, who had injected pus microbes into the peritoneal cavity without producing suppuration, and said that the consensus of opinion seemed to be that for the production of suppuration in the peritoneum, some pathological condition of that membrane must first be present. Grawitz even claims that the injection of intestinal contents into the peritoneum will not cause suppuration so long as the puncture wound remains aseptic.

He concluded by saying that, as clinically, we did not have chemical substances, as turpentine, etc., in the tissues, from a practical point of view it simply was a question of excluding from the wound the pyogenic microbes, which, if present, would produce a substance which would be the direct cause of the suppuration.

DR. W. B. CRAWFORD said, in conclusion, that he had listened with a great deal of pleasure to the discussion evoked by his paper. In reply to Dr. Eve, he said that by asepsis he meant nothing more nor less than perfect cleanliness; that if it was necessary to employ antiseptic agents to bring about this condition, then he unhesitatingly recommended their use. As to the probing of gunshot wounds, aside from the dangers of septic infection from the probing, which, of course, could be eliminated by aseptic precautions, there was produced an irritation by the operation which added unnecessarily to the traumatism already present.

### COUGH OF NASAL ORIGIN.

Dr. Maximilian Herzog reports the case of a young woman in whom an adhesion between the left lower turbinate and the septum had caused for two or three years a most obstinate cough, with the sensation of a foreign body in the throat. Dividing the adhesion with the galvano-cautery knife caused a complete disappearance of these symptoms.—*International Medical Journal.*

### THE HANDS AND IRRITATING ANTISEPTICS.

Dr. Schneider (*Med. Neuigkeiten, No.* 42, 1893), to preserve the hands from being irritated after employing irritating antiseptics, recommends bathing them in alcohol, then washing them off in soap and water, and, while they are still damp and unwiped, rubbing them with lanoline. In case of an irritating solution of corrosive sublimate, bathe them first in a one-half per cent. solution of salt and water and proceed as before.—*Lancet-Clinic.*

